# Illicit cigarette consumption and government revenue loss in Indonesia

**DOI:** 10.1186/s12992-014-0075-7

**Published:** 2014-11-19

**Authors:** Abdillah Ahsan, Nur Hadi Wiyono, Diahhadi Setyonaluri, Ryan Denniston, Anthony D So

**Affiliations:** Demographic Institute Faculty of Economics, University of Indonesia, N. Iskandar Building, 3rd Floor, University of Indonesia Campus, Depok, 16424 Indonesia; Program on Global Health and Technology Access, Sanford School of Public Policy and Duke Global Health Institute, Duke University, Durham, NC 27708 USA

**Keywords:** Illicit, Kretek, Cigarette, Tax loss, Indonesia

## Abstract

**Background:**

Illicit cigarettes comprise more than 11% of tobacco consumption and 17% of consumption in low- and middle-income countries. Illicit cigarettes, defined as those that evade taxes, lower consumer prices, threaten national tobacco control efforts, and reduce excise tax collection.

**Methods:**

This paper measures the magnitude of illicit cigarette consumption within Indonesia using two methods: the discrepancies between legal cigarette sales and domestic consumption estimated from surveys, and discrepancies between imports recorded by Indonesia and exports recorded by trade partners. Smuggling plays a minor role in the availability of illicit cigarettes because Indonesians predominantly consume kreteks, which are primarily manufactured in Indonesia.

**Results:**

Looking at the period from 1995 to 2013, illicit cigarettes first emerged in 2004. When no respondent under-reporting is assumed, illicit consumption makes up 17% of the domestic market in 2004, 9% in 2007, 11% in 2011, and 8% in 2013. Discrepancies in the trade data indicate that Indonesia was a recipient of smuggled cigarettes for each year between 1995 and 2012. The value of this illicit trade ranges from less than $1 million to nearly $50 million annually. Singapore, China, and Vietnam together accounted for nearly two-thirds of trade discrepancies over the period. Tax losses due to illicit consumption amount to between Rp 4.1 and 9.3 trillion rupiah, 4% to 13% of tobacco excise revenue, in 2011 and 2013.

**Conclusions:**

Due to the predominance of kretek consumption in Indonesia and Indonesia’s status as the predominant producer of kreteks, illicit domestic production is likely the most important source for illicit cigarettes, and initiatives targeted to combat this illicit production carry the promise of the greatest potential impact.

## Background

Illicit cigarettes comprise more than 11% of global tobacco consumption and nearly 17% of tobacco consumption in low- and middle-income countries [1]. Increased availability of illicit cigarettes lowers consumer prices through tax evasion. The increased consumption that results from illicit cigarettes threatens both tobacco control efforts and excise tax collection by governments. In the case of Indonesia, illicit cigarettes originate both domestically and abroad. In 1995, an estimated 5% of cigarettes sold in Indonesia were smuggled from abroad, the rest were domestically manufactured cigarettes that were diverted to the black market before taxation [2]. Empirical measurement of the magnitude of illicit cigarette consumption is difficult due to the clandestine nature of the activity. The quality of measurements produced by litter surveys, frequently used to estimate the presence of illicit cigarettes, depend on collection site choices and how well the packs recovered at these sites reflect domestic consumption.

One factor that may fuel illicit trade in the Indonesian market is the presence of a complex tax system, where the excise tax that is levied depends on the type of cigarette produced, the scale of the producing company, the method of production, and the retail price range for the final product. This tax system confers lower excise tax rates to kretek producers over white cigarette producers, to smaller producers over large producers, to producers of hand-rolled kreteks over machine-made cigarettes of either type, and to cheaper final products over more expensive products. In turn, these tax preferences have facilitated the proliferation of numerous, small producers. In contrast to the tobacco industry in nearly every other country, Indonesia possesses a few very large companies and the continued existence of hundreds of small producers, some of which are contracted by the large companies [3].

In 2013, based on Ministry Finance decree No. 179/PMK.011/2012, machine-made kreteks were assessed specific excise taxes of between 50% and 56% of retail price and averaged 53% of retail price. By contrast, hand-rolled kreteks were assessed at between 32% and 37% of retail price and white cigarettes at between 49% and 56% of retail price. Recent changes have reduced tax rate disparities between cigarette tax tiers and raised rates for all tiers, but Indonesia remains a low tax country within the ASEAN region, and excise rates fall below the World Bank’s yardstick of two-thirds to four-fifths of retail price, a range that typifies countries with strong tobacco control efforts and falling consumption [4]. Moreover, roll-your-own (RYO) cigarettes are not taxed if used for personal consumption. A 10% increase in the excise tax would result in an estimated 4% reduction in consumption and a 7-9% increase in tobacco excise revenue [5].

To assess the magnitude of illicit cigarettes in Indonesia, this paper applies two methods. First, survey-based estimates of consumption are compared to cigarette sales. Second, Indonesian cigarette imports are compared to the mirror cigarette exports to Indonesia recorded by trade partners.

The Indonesian market is unique with respect to the dominance of kreteks, a unique tobacco product that is primarily manufactured in Indonesia. The dominance of kreteks among consumers should reduce the importance of cigarettes smuggled from abroad relative to those that originate domestically because most smuggled cigarettes will be white cigarettes. The existence of numerous small producers may also exacerbate the role played by domestic sources in illicit trade [6]. For these reasons, the two estimation methods provide complementary, but different results that help to triangulate in on the magnitude of illicit trade of tobacco. These estimates are produced in a transparent and replicable manner and without the risk of financial conflict of interest inherent in industry-supported studies. These estimates can also provide benchmark comparisons for studies that employ other methods, whether or not produced by the industry.

## Methods

Merriman [7] identified several approaches to estimate the magnitude of illicit cigarettes, three of which are employed by this paper. Of the five methods identified by Merriman, three methods are inexpensive to implement, use publicly available data, and are easily employed without specialized econometric knowledge. First, a comparison of survey-based estimates of consumption to legal tobacco sales directly measures the consumption of illicit cigarettes, irrespective of origin. Consumption of illicit cigarettes is indicated where consumption exceeds sales, which is measured by production less net exports. This method is unable to distinguish illicit cigarettes that originate domestically through multiple channels from those smuggled from abroad, and it can only measure the net magnitude of illicit cigarettes present in a country, which would not accurately represent the true level of illicit trade if large, simultaneous flows into and out of a country offset one another. In addition, under-reporting of smoking behavior by respondents is a documented phenomenon and may produce underestimates of consumption [8-12]. Estimates of the magnitude of under-reporting available in the literature range between 22% for the United States in 1974 and 35% for Italy in 2008. Smoking remains socially acceptable in Indonesia, which would reduce one source for survey respondent under-reporting--the social stigma attached to cigarette smoking. This study estimates the possibility of under-reporting due to faulty recollection and other causes by adjusting consumption by 10%, 20% and 30%.

This study analyzes data from 1995 through 2013 using the estimates of illicit trade produced using tax-paid sales and estimates and 1995 through 2012 using the estimates of illicit trade produced using trade discrepancies. Smoking prevalence and average daily cigarette consumption, used to estimate cigarette consumption, is sourced from several data sources. The National Socio-Economic Survey was used for 1995 and 2004; the Household Health Survey was used for 2001; the Basic Health Research Survey was used for 2007, 2010, and 2013; and the Global Adult Tobacco Survey was used for 2011. Annual population estimates, as well as import and export data, were sourced from the Central Board of Statistics. Cigarette production data were obtained from the Directorate General of Excise and Customs and are based on excise stamp orders. Net exports were deducted from production to obtain tax-paid sales, which are directly compared to consumption.

Second, smuggling into Indonesia is measured by annual trade discrepancies. A trade discrepancy is the difference between exports recorded by the country of origin and imports recorded by Indonesia for a given year, and smuggling into Indonesia is indicated where exports from partners exceed Indonesian imports. The summed discrepancy across all trade partners is an imperfect measure of smuggling activity in that it also captures several legitimate causes for discrepancies including lags between exports and imports, valuation differences due to the inclusion or exclusion of freight and insurance costs, changes in exchange rates, and misreporting of origin where transshipment takes place [13,14]. While the relative importance of these factors is unknown, large and persistent discrepancies would suggest illegal activity [15]. Of note, this method is unable to capture illicit cigarettes that originate within Indonesia and those that evade record keeping at both the origin and destination. Estimates of smuggling will approximate those of consumption of illicit cigarettes only where illicit production is not substantial and will underestimate illicit activity when illicit production is a prominent source for illicit cigarettes. Bilateral trade value data for cigarettes were obtained from the United Nations Commodity Trade Statistics Database and used to measure trade discrepancies. This series differs from the series used for net export data in the first method and is employed both because it captures mirrored exports to Indonesia and because data exists at the bilateral level, which allows for the assessment of especially large sources of smuggled cigarettes. This study chose to use data reported by the Central Board of Statistics for net export data used in the first method because the Central Board of Statistics are the original source for the data found in UN Comtrade. While the UN Comtrade data is collected from governments, small differences emerge with those sources due to changes made by the United Nations as well as revisions to the underlying data. However, the differences between these two trade series are small in magnitude. As a substantial fraction of the country’s tobacco trading partners with Indonesia have not reported trade data for 2013, data are only available through 2012.

To address whether illicit cigarettes are primarily of domestic origin, a third method, the consultation of experts, was employed. The Directorate General of Customs was consulted, and based on this consultation, it is believed that illicit cigarettes are overwhelmingly of domestic origin. This finding suggests that smuggling plays a lesser role in the availability of illicit cigarettes in the Indonesian market and that results based on the first method will produce results that more accurately reflect the context of Indonesia.

Tax losses due to illicit cigarettes were calculated as the product of the estimated volume of illicit cigarettes of domestic origin and the average specific excise tariff applied to domestic cigarettes, plus the product of the estimated volume of smuggled cigarettes and the excise tariff applied to imported cigarettes. Losses are presented for the two most recent survey years, 2011 and 2013. Indonesia currently possesses a tiered tobacco excise system that assesses a specific tax per stick, rather than a percentage of the price. This study uses data that disaggregates cigarette consumption into the three cigarette types and methods of manufacture – machine-made kreteks, machine-made white cigarettes, and hand-made cigarettes of both types. These shares are used to calculate the numbers of illicit cigarettes, by cigarette type. The numbers of cigarettes are in turn multiplied by the highest and lowest tiers for each cigarette type, which provides an upper and lower bound for the estimates of tax losses.

## Results and discussion

Cigarette consumption more than doubled between 1995 and 2013, rising from 136 billion cigarettes to 293 billion cigarettes as shown in Table 1. Smoking prevalence and population both rose over the period. Indonesia’s population increased by nearly 35%, while prevalence increased by 37%, from nearly 27% of the population 15 and over in 1995 to 36.3% in 2013. Smoking intensity rose from 10.6 cigarettes per person per day in 1995 to 12.3 cigarettes in 2013, an increase of 16%. Cigarette production and tax-paid sales both generally rose throughout the period, though both decline between 2001 and 2004. As shown in Table 2, net exports account for between 15% and 21% of domestic production over the period, and this proportion generally rose over time.

**Table 1:** **Smoking prevalence, intensity, and total cigarette consumption, Indonesia, 1995-2013**

**Year**	**Smoking prevalence**	**Population 15 and Over (millions)**	**Intensity (stick/day)**	**Estimated consumption (billion sticks)**
1995	26.9%	131.3	10.6	136.0
2001	31.7%	151.8	11.1	194.1
2004	34.2%	160.5	11.3	227.7
2007	34.2%	166.9	10.2	212.5
2010	34.7%	170.4	10.1	218.0
2011	34.8%	173.5	12.8	282.0
2013	36.3%	179.6	12.3	292.7

Sources: National Socio-Economic Survey 1995 and 2004; Household Health Survey 2001; Basic Health Research Survey 2007, 2010, and 2013; and the Global Adult Tobacco Survey 2011.

Annual consumption is computed as the product of smoking prevalence, the population age 15 and over, daily smoking intensity, and 365 days for the year.

**Table 2:** **Production, sales, and illicit consumption of cigarettes, 1995–2013, billions of sticks**

					**Illicit consumption (under-reporting level)**			
**Year**	**Production**	**Net exports**	**Tax-paid sales**	**Consumption**	**0%**	**10%**	**20%**	**30%**
1995	200.2	29.3	171.0	136.0	−35.0	−21.4	−7.8	5.9
2001	226.6	31.2	195.4	194.1	−1.3	18.1	37.5	56.9
2004	218.6	29.2	189.4	227.7	38.3	61.1	83.9	106.7
2007	241.5	48.1	193.4	212.5	19.1	40.4	61.6	82.9
2010	289.1	54.8	234.3	218.0	−16.3	5.5	27.3	49.1
2011	310.2	58.7	251.5	282.0	30.5	58.7	86.9	115.1
2013	341.9	72.2	269.8	292.7	22.9	52.2	81.4	110.7

Source: Directorate General of Excise and Customs, Ministry of Finance for cigarette productions, Central Board of Statistics for Net Exports Tax-paid sales represent production less net exports. Negative values indicate the absence of illicit cigarette consumption.

The magnitude of illicit cigarette presence in the Indonesian market ranges from −7% to 17% of total consumption for 2004 through 2013 if no respondent under-reporting is assumed to exist, as shown in Table 2. The −7% figure for 2010 represents the only time point where illicit cigarettes are not indicated, and this is followed by an 11% market share for illicit cigarettes in 2011. When any of the under-reporting thresholds are used, illicit cigarettes are also present in 2001 and 2010. Illicit cigarettes consistently increase in magnitude through 2004, fall through 2010, and increase again for 2011 and 2013. This trend parallels that for estimated consumption, that is, both consumption and the presence of illicit cigarettes increase through 2004, fall through 2010, and rise thereafter. Finally, respondent under-reporting may result in an understatement of the magnitude of illicit consumption actually present. When under-reporting is assumed to be 30%, the magnitude of illicit cigarettes peaks at 47% of domestic consumption in 2004.

Smuggling as measured by summed, annual trade discrepancies across all trade partners is sizable relative to both imports recorded by Indonesia and exports recorded by trade partners. As shown in Table 3, discrepancies generally surpass 90% of total trade, the sum of Indonesian imports and exports to Indonesia, and amount to between less than one million and almost 30 million US dollars in smuggling activity per year. Smuggling declined sharply between 1995 and 2000 before increasing through the remainder of the period. In 2011, smuggling peaked at $49 million. However, annual discrepancies as a share of total trade fell after 2007. Direct comparison of these results to those of consumption of illicit cigarettes is not possible due to the unavailability of quantity-based trade data. However, while the trade discrepancies are large compared with recorded Indonesian imports, imports represent a small fraction of domestic consumption, and smuggling is considered a relatively small source for illicit cigarettes available within the Indonesian market.

**Table 3:** **Indonesian cigarette imports and trade discrepancies, 1995–2012, in millions of US dollars**

**Year**	**Indonesian imports**	**Exports to Indonesia**	**Trade discrepancy**	**Discrepancy as share of total trade (%)**
1995	1.5	28.8	27.3	90.0
1996	.7	24.2	23.5	94.8
1997	.6	2.9	2.3	64.3
1998	.3	6.0	5.8	89.3
1999	.6	2.7	2.1	62.4
2000	1.7	2.3	.6	15.1
2001	.6	5.4	4.8	80.7
2002	.2	6.5	6.3	94.3
2003	.1	16.6	16.5	98.3
2004	.2	21.4	21.3	98.4
2005	1.0	26.5	25.5	92.7
2006	.5	30.2	29.8	96.8
2007	.4	38.2	37.8	97.7
2008	2.5	44.8	42.4	89.6
2009	2.8	36.6	33.7	85.7
2010	4.9	45.0	40.1	80.4
2011	5.2	54.4	49.2	82.4
2012	11.0	58.7	47.7	68.3

Source: United Nations Commodity Trade Statistics (Comtrade) database.

Total trade is the sum of imports recorded by Indonesia and exports to Indonesia recorded by trade partners.

Singapore, China, and Vietnam together represent the origin for more than 64% of tobacco trade discrepancies over the entire period, with Singapore accounting for 28%, as shown in Table 4. The importance of particular countries as origins for smuggled cigarettes changed over time. Specifically, discrepancies with Hong Kong fell sharply after 1995, while discrepancies with China fluctuated over the period. Singapore and Vietnam both rose in importance in the mid-2000s. Singapore was the largest source for smuggled cigarettes in 2004 and afterwards, accounting for more than one-third of smuggled cigarettes in the last decade. The five identified countries in Table 4 account for more than 85% of Indonesia’s net trade discrepancy with the world over the period.

**Table 4:** **Trade discrepancies among top 5 exporters of cigarettes to Indonesia, 1995–2004, selected years**

**Partner**	**1995**	**1998**	**2001**	**2004**	**2007**	**2010**	**2012**	**Discrepancy share, 1995–2013 (%)**
Singapore	−1.3	0	0	14.3	13.3	12.1	14.9	27.8
China	4.6	5.5	1.1	3.9	6.5	7.4	7.3	19.3
Vietnam	0	0	0	.1	4.2	11.1	18.6	17.2
Hong Kong	21.2	.3	0	.2	5.0	.7	-.3	16.1
Malaysia	2.8	.1	1.8	.5	.6	2.7	.9	5.5
Others	0	.2	.7	2.4	8.3	6.1	6.3	14.0
World	27.3	5.8	4.8	21.3	37.8	40.1	47.7	

Source: United Nations Commodity Trade Statistics (Comtrade) database.

Two factors complicate the estimation of excise revenue losses. First, while the majority of cigarettes consumed are kreteks, most smuggled cigarettes are white cigarettes. White cigarettes are assessed different tax rates than kreteks, as noted above. The experience of the Directorate General of Customs and Excise officer consulted for this study indicated that illegal domestic production far exceeds cigarette smuggling, and illicit production comprised roughly 90% of illicit cigarettes. Second, Indonesia’s tiered tax system conveys a different assessed tax level on each cigarette, which depends on the cigarette type, the size of the production facility, the method of manufacture, and the retail price. While the proportions of cigarettes of each cigarette type are known, the proportions that fall within each tax tier are not. Machine-made kreteks account for 68.5% of domestic consumption, hand-made kreteks and white cigarettes for another 25.5%, and machine made white cigarettes for the remaining 6%. As each of these types contains a different set of tax tiers, exact estimation of the tax losses is not possible.

Tax losses are presented as ranges that denote the upper and lower bounds of tax losses given the assessed rates, distribution of consumed cigarettes among cigarette types, and the proportion of illicitly consumed cigarettes that are smuggled into Indonesia. Table 5 presents two ranges for both 2011 and 2013, one where no illicit cigarettes are assumed to originate abroad, and one where 10% of illicit trade is assumed to originate abroad. For each range, the lower bound is the sum of the numbers of cigarettes falling into each cigarette type multiplied by the lowest tax tier for that cigarette type. The upper bound uses the highest tax rate assessed for each type of cigarette. For 2011, losses range between Rp 4.1 trillion and Rp 9.3 trillion, or between 5.6% and 9.3% of tobacco excise collections. Losses in 2013 range from Rp 4.5 and Rp8.1 trillion, or 4.3% to 7.8% of tobacco excise collections.

**Table 5:** **Government revenue loss due to illicit cigarettes (assuming no under-reporting) Indonesia, 2011 and 2013**

**Category**	**Subcategory**	**2011**		**2013**	
Origin of Illicit Consumption (%)	Illicitly Produced	90	100	90	100
	Smuggled	10	0	10	0
Illicit Cigarettes (billion sticks)	Illicitly Produced	27.4	30.5	20.6	22.9
	Smuggled	3.0	0	2.3	0
Proportion of Domestic Consumption (%)	Machine Made Kreteks Cigarette	68.5%	68.5%	68.5%	68.5%
	Machine Made White Cigarettes	6.0%	6.0%	6.0%	6.0%
	Hand-Made Kreteks and White Cigarettes	25.5%	25.5%	25.5%	25.5%
Lowest Tax Tiers (Rp)	Machine Made Kreteks	170	170	245	245
	Machine Made White Cigarettes	110	110	195	195
	Hand-Made Kreteks and White Cigarettes	65	65	80	80
Highest Tax Tiers (Rp)	Machine Made Kreteks	325	325	375	375
	Machine Made White Cigarettes	325	325	380	380
	Hand-Made Kreteks and White Cigarettes	235	235	275	275
Estimated Revenue Loss Range (Rp)	Estimate, Lowest Tier (trillion Rp)	4.1	4.3	4.5	4.6
	Estimate, Highest Tier (trillion Rp)	9.3	9.2	8.1	8.0
Estimated Revenue Loss Range (US$)	Estimate, Lowest Tier (million US$)	467.5	490.3	430.2	439.7
	Estimate, Highest Tier (million US$)	1,060.4	1,049.0	774.3	764.7
Estimated Revenue Loss Range as Share of Tobacco Excise Taxes	Total Tobacco Excise Revenue (trillion Rp)	73.3	73.3	103.6	103.6
	Share of Tobacco Excise Revenue, Lowest Tier (%)	5.6	5.9	4.3	4.4
	Share of Tobacco Excise Revenue, Highest Tier (%)	12.7	12.6	7.8	7.7

Source: Authors’ calculations.

Official exchange rates were Rp 8770.4 per US$ in 2011 and 10,461.2 in 2013.

Estimated tax losses were most sensitive to the differences between highest and lowest tax tiers applied to each type of cigarette, as opposed to changes in the level of smuggled cigarettes. In 2011, the tax losses change from Rp 4.3 trillion to Rp 4.1 trillion when the assumed proportion of cigarettes smuggled rises to 10% at the lowest tax tiers, while at the highest tiers, the losses rise from Rp 9.2 to Rp 9.3 trillion. By contrast, the key driver of tax losses is the difference in tobacco tax tiers. If no smuggling takes place, tax losses range between Rp 4.3 and Rp 9.2 trillion, whereas if smuggling comprises 10% of illicit cigarettes, tax losses range between Rp 4.1 and Rp 9.3 trillion.

## Conclusion

By its nature, estimation of the magnitude of illicit cigarettes must involve triangulation by several methods. This paper employs three of Merriman’s proposed methods to estimate illicit trade and to assess the relative contributions of illegally produced and smuggled cigarettes to illicit trade. Customs and excise officials identified illicit production as the primary source of illicit cigarettes, responsible for at least 90% of illicit cigarettes. This suggests that estimates based on the comparison of tax-paid sales to consumption as estimated by surveys will differ substantially from those produced by the measurement of trade discrepancies, and that the former will represent an estimate that more fully captures the magnitude of illicit trade in Indonesia. Although survey respondents may under-report consumption, intentional under-reporting is not thought to be a significant problem in the Indonesian context due to the social acceptability of smoking among Indonesian men, the predominant consumers of cigarettes [16]. By contrast, trade discrepancies, while useful, will only capture a small fraction of available illicit cigarettes in the Indonesian market. Most illicit cigarettes are thought to be illegally produced within Indonesia because Indonesia is the primary producer and consumer of kreteks.

Before 2004, Indonesia reported more legal sales than consumption, possibly due to a combination of respondent under-reporting and smuggling out of cigarettes to other countries. When respondent under-reporting is not assumed to exist, the consumption of illicit cigarettes first emerges in 2004 as measured by the comparison of sales and survey-based estimates of consumption. With the exception of 2010, the consumption of illicit cigarettes is present between 2004 and 2013. Illicit cigarettes amounted to between 19 billion and 38 billion sticks per year, or between 8% and 17% of total consumption per year. These illicit cigarettes cost the Indonesia government at least Rp 4.1 trillion in 2011 and Rp 4.5 trillion in 2013, at least 5.6% and 4.3% of total tobacco excise revenue respectively. Put differently, illicit trade cost the government between $468 million and more than $1 billion in 2011 and between $430 million and $774 million in 2013.

Importantly, the emergence of illicit cigarettes exists within a context where cigarette consumption, smoking prevalence, intensity, and the population of Indonesia rose. Cigarette consumption more than doubled between 1995 and 2013 to an estimated 293 billion cigarettes by 2004, as shown in Table 1. Increased population and increased prevalence are roughly equal in importance to increased consumption. Having not ratified the Framework Convention on Tobacco Control, Indonesia also has low cigarette prices relative to other countries in the region and lags behind many other countries with respect to the enactment of strict tobacco control policies. These factors may play a role in the increase in cigarette consumption [3,17,18]. While cigarette production and cigarette exports both rose over time, production did not increase enough to account for the increases in both consumption and net exports. This suggests that illicit trade may be increasing.

Two unique characteristics of tobacco consumption in Indonesia may facilitate a predominance of domestic origin for the illicit trade. First, the predominance of kretek consumption in Indonesia suggests that smuggled cigarettes cannot account for all illicit consumption, as Indonesia is the predominant manufacturer of kreteks. Second, the structure of the Indonesian tobacco industry may unintentionally and indirectly facilitate illicit production. Indonesia houses thousands of cigarette producers, many of which go unregistered. In Jember District of East Java Province alone, there were 242 cigarette producers; most of them (213 cigarette producers) did not possess identification numbers as required by the Excise Tax Directorate [19]. If costs to effectively monitor such a large number of producers are prohibitively high, illicit activity may take place with less risk of detection. This structure coexists with a tiered tax system that favors small producers and producers of hand rolled kreteks.

While smuggling was not a predominant source for illicit cigarettes, Indonesia was a consistent recipient of smuggled cigarettes, most likely white cigarettes. Hong Kong was the primary source of smuggled cigarettes, but its importance has been replaced by China and Singapore. In fact, one newspaper report states that between 2004 and 2005, the Directorate General of Excise and Customs intercepted 6.8 million packs of cigarettes originating in China valued at 20 billion rupiah [20]. However, white cigarettes comprise a small share of the domestic market and illicit consumption.

Recent policy developments and efforts by the Ministry of Finance have reduced the favorable conditions received by small producers and have actively facilitated the exit of numerous small producers. Ministry of Finance decree No. 200/PMK.04/2008, issued in 2008, stipulated that tobacco manufacturers must possess at least 200 square meters of physical plant area to operate. In addition, this physical plant must not be directly related to a residence or a space not related to the tobacco manufacturer license. This has the effect of reducing the numbers of small producers and manufacturers which produce cigarettes as a supplementary activity to principal business operations. In addition, several tobacco excise tax increases enacted by legislation No. 190.PMK.011/2010 and subsequent amendments have not only raised the proportion of retail value subject to excise tax, but have reduced the numbers of tax tiers in a manner that has narrowed the tax gap between expensive and inexpensive cigarettes and kreteks [21,22]. Third, legislation No. 191/PMK.04/2010 eliminated the ability of large manufacturers to produce cigarettes at small facilities that are established or acquired by the parent company, which benefit from lower assessed rates. Instead, cigarettes produced at these small facilities will be assessed the rate at which the parent company is assessed, which reduces the taxes that can be avoided through funneling of production through small producers [21]. Finally, increased monitoring from tax authorities and competition from large manufacturers reduced the numbers of producers from as many as 4,700 in 2007 to 3,800 in 2010, 1,700 in 2011, and as few as 1,000 by the end of 2012 [23]. If illicit cigarettes of domestic origin primarily originate from small producers, these efforts should reduce the availability of illicit cigarettes in the future.

To further reduce the illicit production and smuggling of cigarettes, the government needs to enforce existing law, strictly monitor producers, and impose penalties. These efforts will produce substantial tax revenue and will improve health and life expectancy in Indonesia. Limitation of duty free sales, imposition of regulation to mandate use of more effective tax stamps, and license of all parties involved in the production and distribution of tobacco products may also reduce illicit trade and the availability of illicit cigarettes. Finally, targeted efforts in cooperation with principal sources for smuggled cigarettes may be more cost effective than broad-based efforts to reduce smuggling where resources are limited. As Indonesia almost certainly serves as an origin for smuggled kreteks to other countries in the region, targeted efforts may also reduce the availability of illicit cigarettes among these trade partners.
